# Diagnosis of idiopathic normal pressure hydrocephalus is supported by MRI-based scheme: a prospective cohort study

**DOI:** 10.1186/1743-8454-7-18

**Published:** 2010-10-31

**Authors:** Masaaki Hashimoto, Masatsune Ishikawa, Etsuro Mori, Nobumasa Kuwana

**Affiliations:** 1 Department of Neurosurgery, Noto General Hospital, Nanao, 926-8610, Japan; 2 Department of Neurosurgery, Rakuwakai Otowa Hospital, Kyoto, 607-8602, Japan; 3 Department of Behavioral Neurology and Cognitive Neuroscience, Tohoku University Graduate School, Sendai, 980-8575, Japan; 4 Department of Neurosurgery, Tokyo Kyosai Hospital, Tokyo, 153-8934, Japan

## Abstract

**Background:**

Idiopathic normal pressure hydrocephalus (iNPH) is a treatable neurological syndrome in the elderly. Although the magnetic resonance imaging (MRI) findings of tight high-convexity and medial subarachnoid spaces and the ventriculo-peritoneal (VP) shunt with programmable valve are reportedly useful for diagnosis and treatment, respectively, their clinical significance remains to be validated. We conducted a multicenter prospective study (Study of Idiopathic Normal Pressure Hydrocephalus on Neurological Improvement: SINPHONI) to evaluate the utility of the MRI-based diagnosis for determining the 1-year outcome after VP shunt with the Codman-Hakim programmable valve.

**Methods:**

Twenty-six centers in Japan were involved in this study. Patients aged between 60 and 85 years with one or more of symptoms (gait, cognitive, and urinary problems) and MRI evidence of ventriculomegaly and tight high-convexity and medial subarachnoid spaces received VP shunt using the height/weight-based valve pressure-setting scheme. The primary endpoint was a favorable outcome (improvement of one level or more on the modified Rankin Scale: mRS) at one year after surgery, and the secondary endpoints included improvement of one point or more on the total score of the iNPH grading scale. Shunt responder was defined by more than one level on mRS at any evaluation point in one year.

**Results:**

The full analysis set included 100 patients. A favorable outcome was achieved in 69.0% and 80.0% were shunt responders. When measured with the iNPH grading scale, the one-year improvement rate was 77.0%, and response to the surgery at any evaluation point was detected in 89.0%. Serious adverse events were recorded in 15 patients, three of which were events related to surgery or VP shunt. Subdural effusion and orthostatic headache were reported as non-serious shunt-related adverse events, which were well controlled with readjustment of pressure.

**Conclusions:**

The MRI-based diagnostic scheme is highly useful. Tight high-convexity and medial subarachnoid spaces, and enlarged Sylvian fissures with ventriculomegaly, defined as disproportionately enlarged subarachnoid-space hydrocephalus (DESH), are worthwhile for the diagnosis of iNPH. This study is registered with ClinicalTrials.gov, number NCT00221091.

## Background

Normal-pressure hydrocephalus (NPH) is well known as a treatable syndrome of the classical triad of gait disturbance, dementia, and urinary disturbance [1], and can be classified into idiopathic NPH (iNPH) and secondary NPH [2]. It develops in elderly people, and the rapid increase in the number of elderly people among the general population in Japan prompted the Japanese Society of Normal Pressure Hydrocephalus to formulate clinical guidelines for iNPH [3,4]. The Japanese Guidelines were published in 2004 in Japanese [3], and the English version of them was published in 2008 [4], and the international guidelines for the diagnosis and management of iNPH have now also been published [5-9]. These guidelines played an important role in making more widely known the diagnosis and management of iNPH. However, there remain unanswered questions in relation to both the diagnosis and treatment of iNPH.

Although tightness of high-convexity and medial subarachnoid spaces, which is most appreciable on coronal MRI sections, is reportedly a characteristic feature of iNPH [10] and is recommended as a supportive sign of the condition in the Japanese guidelines, its diagnostic value remains to be validated in a large number of suspected iNPH patients. Furthermore, although ventriculo-peritoneal (VP) shunt with a programmable valve is recommended for treating hydrocephalus in general [11,12], and for treating iNPH in the Japanese guidelines [3,4], the outcome of iNPH patients who received VP shunt with a programmable valve remains to be determined [13]. We conducted a multicenter prospective study (Study of Idiopathic Normal Pressure Hydrocephalus on Neurological Improvement: SINPHONI) to evaluate the utility of the MRI-based diagnosis for determining outcome 1-year after VP shunt with Codman-Hakim programmable valve (CHPV). Herein, we report the general profile of SINPHONI and its primary findings.

## Methods

### Study design

The study was a multicenter prospective cohort study conducted in compliance with the Guidelines for Good Clinical Practice and the Declaration of Helsinki (2002) of the World Medical Association. The study protocol was approved by the institutional review board at each site, and all patients (or their representatives when applicable) gave written informed consent for participation. Twenty-six centers in Japan were involved in this study (see Acknowledgement). The first patient was recruited on November 8, 2004, and the follow-up of the last patient was completed on December 28, 2006. The Translational Research Informatics Center (TRI-Kobe, Japan) together with the steering committee, monitored all the clinical data, imaging data, data related to safety issues, and protocol compliance via the web-based case report system. This study is registered with ClinicalTrials.gov, number NCT00221091.

### Participants and protocol

**Figure 1:**
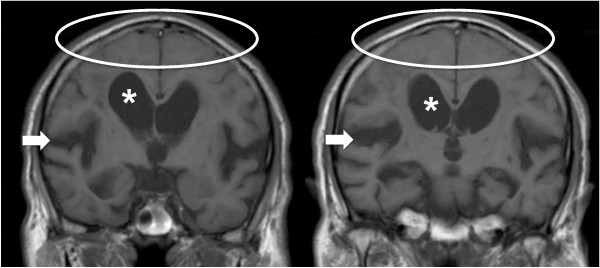
**Typical iNPH findings on MRI**. Illustrative coronal sections of coronal T1-weighted images selected from an included patient showing enlarged ventricles (*), tight high-convexity and medial surface subarachnoid spaces (oval ring), and expanded Sylvian fissures (arrow).

The candidates for this study were patients with suspected iNPH. After obtaining written informed consent, the eligible patients were pre-registered and received lumbar puncture. The inclusion criteria were (1) age between 60 and 85 years, (2) presence of one or more symptom(s) of the triad (gait disturbance, cognitive impairment, and urinary symptoms), which were measurable on the iNPH Grading Scale (iNPHGS) [14], (3) MRI features of iNPH, i.e., both ventriculomegaly of Evans' index > 0.3 and tight high-convexity and medial subarachnoid spaces on coronal T1-weighted MRI (Figure 1) [10], (4) absence of known disorders causing ventriculomegaly, and (5) normal cerebrospinal fluid (CSF) content (protein ≤ 50 mg/dl and cell count ≤ 3 μm^3^) and pressure (≤ 20 cmH_2_O). Exclusion criteria were (1) presence of musculoskeletal, cardiopulmonary, renal, hepatic, or mental disorders that would make it difficult to evaluate changes of symptoms, (2) obstacles to one-year follow-up, and (3) hemorrhagic diathesis or anticoagulant medication. For the evaluation of the MRIs, Evans' index, size of the Sylvian fissures rated according to the protocol of Kitagaki *et al. *[10], presence or absence of focal dilatation of the cerebral sulci, and white-matter changes according to scale of Fazekas *et al. *[15], were assessed on each site and recorded.

The candidates were pre-registered before CSF examination via a web-based case report system. MRI was reviewed by each site in the pre-registration phase, and the final eligibility of the subjects was judged by the central MRI review committee, which consist of neurosurgeons, neurologists, and a neuroradiologist. The central MRI review committee excluded those whose MRI did not fulfil the inclusion criteria. After the confirmation of normal CSF content and pressure, the investigator was notified of registration via the web system. Tap test was carried out in all subjects with 30 ml CSF removal via lumbar puncture. CT cisternography was carried out 1 week after the tap test with iohexol (Omnipaque^®^: 180 mg/ml) 30 mg/kg. Cerebral blood flow was measured using ^123^I-Iodoamphetamine and single photon emission computed tomography at baseline. However, the results of these measures were not considered for the eligibility.

### Outcome measures

The primary endpoint was improvement of one level or more on the modified Rankin Scale (mRS) [16] at one year after surgery (favorable outcome). The secondary outcome measures included the iNPHGS [14], timed 3 meter "Up & Go" test (TUG) [17], and the Mini-Mental State Examination (MMSE) [18]. The follow-up also included CT scan. These measures were repeated at baseline and at 3, 6, and 12 months after shunt surgery. A positive response to VP shunt (shunt responder) was defined as improvement by more than 1 level on mRS at any evaluation point in one year.

VP shunt was carried out under general anesthesia using a shunt system with CHPV. The initial pressure for the shunt system was set before surgery according to a standardized scheme, in which the initial pressure setting was calculated from the patients' height and body mass index based on the estimated hydrostatic pressure and intra-abdominal pressure [19,20]. The valve pressure was readjusted by 1-3 cmH_2_O interval afterward as required. Shunt function was assessed at each site whenever needed. If a surgical intervention was made to repair the shunt, adverse event report was required as described below. Safety assessments included the recording of all adverse events throughout the study, irrespective of whether or not they were suspected to bear any relation to treatment. A serious adverse event (SAE) was defined as one of the followings: (1) death, (2) an event that could result in death, (3) an event for which treatment requires hospitalization or admittance to a clinic or an extension of the hospitalization period, and (4) an event that could result in morbidity.

### Statistical analysis

To detect a 50% rate for favorable outcome, which was considered as a clinically meaningful benefit, with a 95% confidence interval (CI) of 40-60%, it was calculated that 97 patients are necessary. Therefore, to allow for any withdrawals from the study, the sample size was set at 110. Analyses were based on the full analysis set. Where there was missing data at any follow-up time point, data was imputed using the "last observation carried forward". When no follow-up data was available, the maximally unfavorable score was allocated. The changes from baseline to one year post-surgery were analyzed on the mRS, iNPHGS, TUG, and MMSE using Wilcoxon test in terms of total subjects, and subgroups with favourable and unfavourable outcome. Descriptive statistics were expressed as the mean and standard deviation (SD) or as the median and 25-75 percentile interval.

## Results

### Trial profile

**Figure 2:**
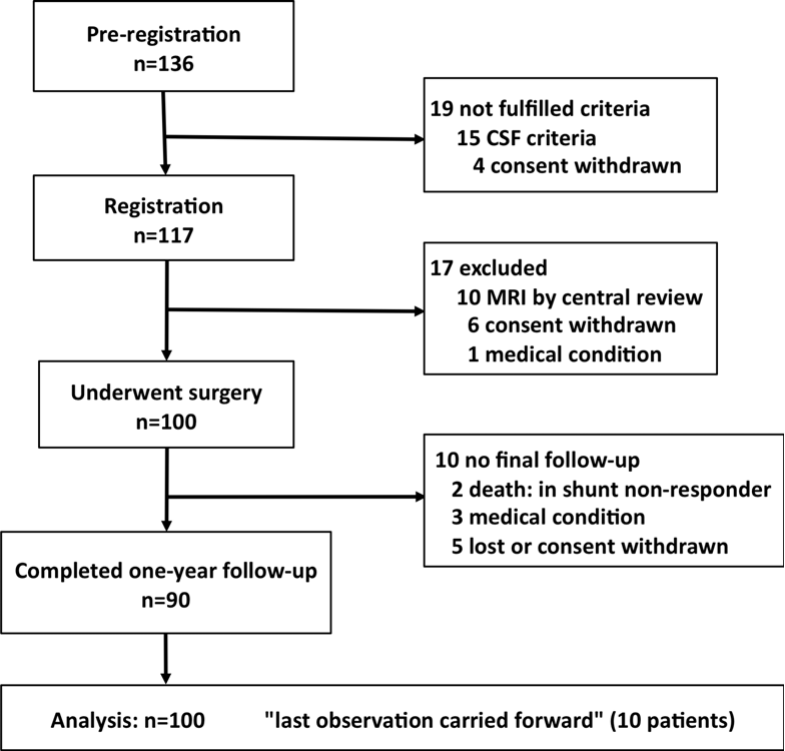
**Trial profile showing flow chart of patient numbers from initial screening to final analysis**.

**Figure 3:**
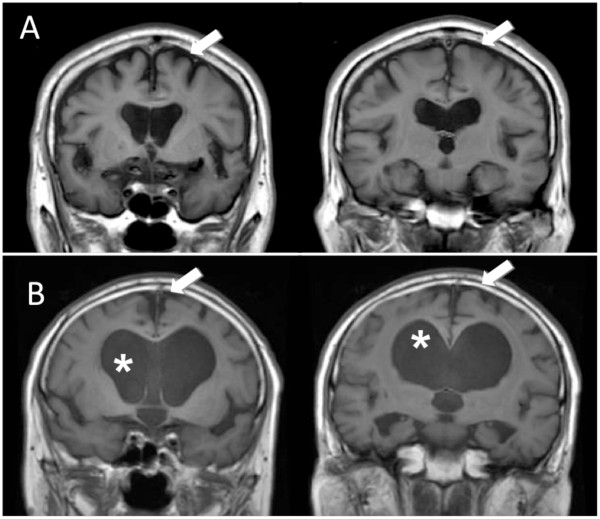
**Two cases excluded by the central MRI review**. MRIs of two illustrative cases excluded by the central MRI review board because of deviation from the pre-set MRI criteria. Note the enlarged subarachnoid spaces (arrow) proportionate to ventriculomegaly indicating brain atrophy (A), and the open high-convexity and medial subarachnoid spaces (arrow) despite disproportionately expanded ventricles (*) suggesting chronic occlusive hydrocephalus (B).

The flow chart for this study is shown in Figure 2. Pre-registration was completed for 136 patients. After lumbar puncture, 15 patients were withdrawn because of failure to fulfil the CSF inclusion criteria. Four patients withdrew their consent between the pre-registration and registration. Thus, 117 patients proceeded to the registration. At this stage, the central MRI review committee excluded ten patients because of deviation from the MRI criteria: eight patients lacked tight high-convexity and medial subarachnoid spaces (Figure 3) and two patients had intracranial mass lesions. Six patients who subsequently withdrew their consent and one who had pulmonary tuberculosis did not receive surgery. Thus, the full analysis set comprised the remaining 100 patients. Ten patients failed to receive one-year final follow-up (death: 2, cerebral infarction: 1, myocardial infarction: 1, femoral fracture: 1, and lost or withdrawal of consent: 5), so that the follow-up completion rate was 90%.

### Patients' characteristics and MRI signs

**Figure 4:**
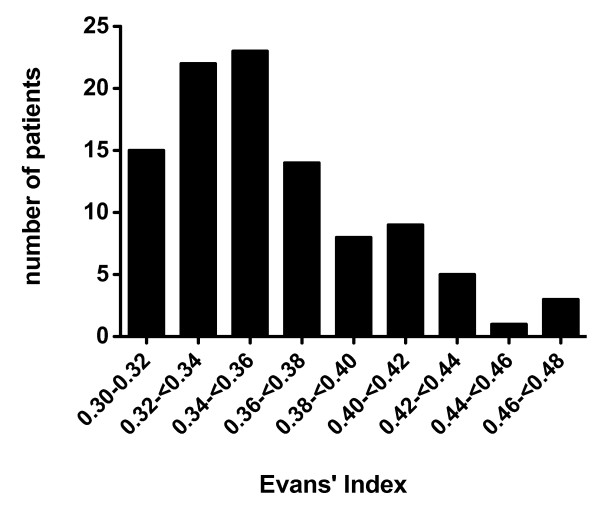
**Bar chart showing the distribution of Evans' index in the patient population**. The mean Evans' index was 35.6 ± 4.0% ranging 0.30 to 0.48, which indicated that the dilation of lateral ventricle in this group was mild to moderate, and marked dilation was rare.

**Figure 5:**
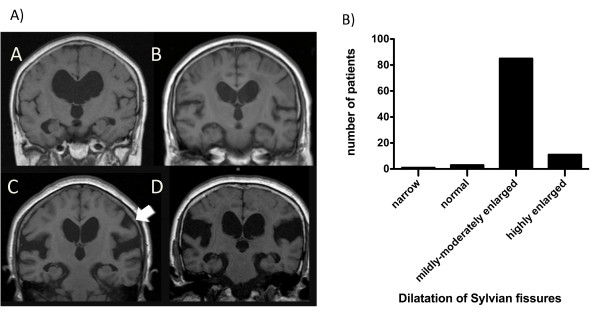
**Dilatation of Sylvian fissures**. A) Illustrative MRI sections showing the variable size of Sylvian fissure. A: narrowing, B: normal, C: mildly-moderately enlarged, D: highly enlarged, A (C): Arrow indicating focal sulcal dilatation. B) Bar chart showing patient distribution of the four dilation categories described above. The Sylvian fissures were dilated in 96% of patients.

The demographic characteristics of the full analysis set are summarized in Table 1. The mean age was 74.5 ± 5.1 (SD) years, and there were 58 men and 42 women. Objective symptoms of severity of 2 or more on the iNPHGS were noted in 91% for gait disturbance, in 80% for cognitive impairment, and in 60% for urinary symptoms. The combination of symptoms is listed in Table 2. The classical triad was noted in 51%. The mean CSF pressure on lumbar puncture was 11.9 ± 3.4 cmH_2_O. On MRI, the mean Evans' index was 35.6 ± 4.0%; the distribution of Evans' index was illustrated in Figure 4. Classification of the size of Sylvian fissures is illustrated in Figure 5A (A-D). Focal dilatation of the cerebral sulci was noted in 29%, Figure 5A (C) arrow. Overall the Sylvian fissures were dilated in 96% (Figure 5B). Mild/moderate and severe white-matter changes were present in 52% and 14%, respectively.

**Table 1:** Background characteristics for patients in study

Age (years)	74.5 ± 5.1
Sex (male)	58%
Education (years)	10.2 ± 2.9
Systolic pressure (mmHg)	131.0 ± 14.6
Diastolic pressure (mmHg)	75.7 ± 8.9
History of hypertension	60%
History of diabetes	20%
History of a lipid disorder	31%
Smoking status	
Currently	10%
Previously	37%
Medications (% present)	
L-dopa/dopamine agonist	10%
Donepezil	6%
Antipsychotics	3%
Antidepressants	8%
Antiplatelet agents	18%
Antihypertensives	49%
Antidiabetic agents	13%
Lipid-lowering medication	11%

Data are % present or mean ± standard deviation.

**Table 2:** Combination of symptoms

Combination of symptoms*	No. of patients
Triad	51
Gait and cognitive	23
Gait and urinary	5
Cognitive and urinary	3
Gait only	12
Cognitive only	3
Urinary only	1
Subjective symptoms only	2

* Objective symptoms of score ≥ 2 on the idiopathic normal pressure hydrocephalus grading scale (iNPHGS) were listed, and subjective symptoms imply symptoms of score = 1 on the iNPHGS.

### Clinical outcomes

**Figure 6:**
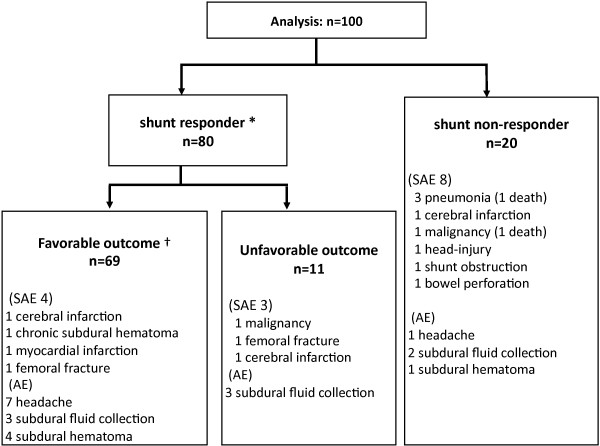
**Flow chart showing the outcome for all patients analysed**. There were 80 shunt responders with 69 having a favourable outcome. There were 20 non-responders, 12 of which had some adverse event. Serious adverse events are listed. * Functional improvement (≥ 1 improvement on mRS) at some point during one year. † Functional improvement (≥ 1 improvement on mRS) at one year. mRS: modified Rankin Scale. SAE: Serious adverse event. AE: Adverse event.

**Figure 7:**
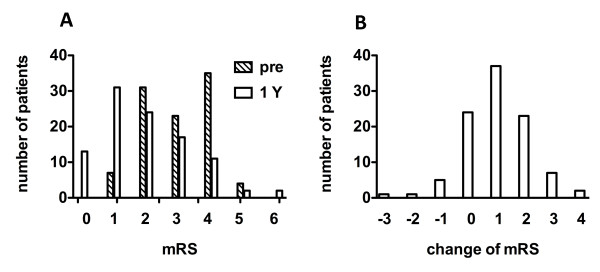
**Bar charts of patients' functional status based on the modified Rankin scale (mRS)**. A: Distribution at baseline and one year. B: Change between baseline and one year.

Shunt surgery was successfully completed in all patients. The mean initial valve pressure was 15.5 ± 4.3 cmH_2_O, and was maintained throughout the follow-up period in 46 patients and readjusted in the rest. A favorable outcome was achieved in 69%, 95% CI: 59.4-77.2% (Figure 6). The distributions of the patients' functional status at baseline and one year and their changes are illustrated in Figure 7A, B. The number of patients without functional impairment (mRS ≤ 1) increased from 7 at baseline to 44 at one year; the number of independent patients (mRS ≤ 2) increased from 38 at baseline to 68 at one year. Scores on the iNPHGS, TUG, and MMSE are summarized with each outcome group in Table 3. All the secondary outcome measures significantly improved in the analysis of all the patients and in the analysis of the subgroup with favorable outcome. Even in the analysis of the subgroup with unfavorable outcome, a significant improvement was noted in time on the TUG. When one point or more decrement on the total score of the iNPHGS is regarded as clinical improvement, benefits were noted in 77% of the subjects at one year after surgery. Shunt responder (improvement ≥ 1 on mRS at any evaluation point in one year) was noted in 80%, 95% CI: 71.0-86.7% (Figure 6). When using the iNPHGS as a measure, the response to the surgery was detected in 89% of the subjects.

**Table 3:** Changes of outcome measures with each outcome group.

	Baseline	12 months	*p *value*
INPHGS gait score			
All patients	2 (2.3-2.6)	1 (1.2-1.7)	< 0.001
Favorable outcome	2 (2.3-2.6)	1 (0.8-1.3)	< 0.001
Unfavorable outcome	2 (2.1-2.7)	2 (1.9-2.8)	ns

iNPHGS cognitive score			
All patients	2 (2.1-2.4)	1 (1.3-1.7)	< 0.001
Favorable outcome	2 (2.0-2.5)	1 (1.0-1.5)	< 0.001
Unfavorable outcome	2 (1.8-2.6)	2 (1.6-2.4)	ns

iNPHGS urinary score			
All patients	2 (1.7-2.2)	1 (0.8-1.3)	< 0.001
Favorable outcome	2 (1.5-2.1)	1 (0.5-0.9)	< 0.001
Unfavorable outcome	2 (1.8-2.8)	2 (1.4-2.4)	ns

iNPHGS total score			
All patients	6 (6.1-7.1)	4 (3.4-4.6)	< 0.001
Favorable outcome	6 (5.9-7.1)	2 (2.4-3.6)	< 0.001
Unfavorable outcome	6 (5.9-7.9)	5 (5.1-7.5)	ns

Timed "up & go" test (seconds)			
All patients	20.5 (16-30)	14 (11-20)	< 0.001
Favorable outcome	20 (16.5-28)	13 (10-17)	< 0.001
Unfavorable outcome	22 (15-41)	20 (13-33)	0.004

MMSE			
All patients	23 (16.3-26)	25 (20.3-28)	< 0.001
Favorable outcome	23 (15.5-26)	25 (21.5-28)	< 0.001
Unfavorable outcome	23 (17-25)	24 (16-27)	ns

Data are median (75th percentile).

* All *p *values were obtained using Wilcoxon test. ns: not significant.

iNPHGS: Idiopathic Normal-Pressure Hydrocephalus Grading Scale, MMSE:Mini-Mental State Examination.

### Adverse events

Serious adverse events (SAE) were noted in 15 patients (Figure 6): death occurred in two patients (lung cancer and pneumonia). Outcome at one year was favourable in 4 patients and unfavourable in 11 patients (including the two deaths). Three SAEs were directly related to surgery or the VP shunt: subdural hematoma requiring surgery, bowel perforation requiring surgical repair, and ventricular shunt tube obstruction requiring revision. In addition, 21 non-serious shunt-related adverse events were recorded in 20 patients: asymptomatic subdural effusion on brain imaging in 13 patients and postural headache in 8 patients, which were successfully controlled in all cases by adjustment of valve pressure.

## Discussion

The present study examined both the usefulness of the MRI-based diagnostic scheme and the one-year beneficial effect of shunt surgery for patients with iNPH. The diagnosis of iNPH is established in terms of response to surgery, and the efficacy of surgery depends on diagnostic accuracy. Therefore, there is an interaction between the accuracy of the diagnostic scheme and the efficacy of the treatment. Nevertheless, we achieved an improvement rate of 69.0% on daily life activity, which indicates that all the procedures, including diagnosis and treatment, yield reasonable net benefits. Furthermore, we proved that the diagnostic scheme has a high positive predictive value, as the percentage of subjects who were found at one evaluation point to have responded to the VP shunt was 80.0% for the whole group, which indicates that MRI-based diagnosis is useful for the diagnosis of iNPH.

Assessment of the outcome in iNPH patients is an important issue; activity of daily life would be more practical for assessing surgical outcome, and NPH symptoms would be better for the shunt efficacy. In this study, clinical improvement was defined as at least a one level improvement on mRS. The mRS is the most popular assessment of global outcome in stroke [16], and has also been used to measure disability in other non-stroke neurological diseases [21-23], including iNPH [24,25]. As the grading definitions of the mRS are very broad classifications of disability, only sizable changes can be detected [22]. Moreover, inter-rater reliability may be an important source of error with the mRS. However, in this study, improvements were detected in a large proportion of the subjects, suggesting a significant treatment effect. The improvements were also confirmed on all the other scales used. In particular, the iNPHGS, which is a valid and reliable scale specific for iNPH [14], provided a very sensitive means of detecting change; this scale detected improvements in 77.0%. The timed "Up & Go" test also detected improvements even in the subgroup with unfavorable outcome, where no improvement or deterioration was recorded on the less sensitive mRS. Therefore, if the low sensitivity of the mRS is taken into consideration, the outcome in this study is acceptable.

Serious adverse events occurred in 15 patients, and all but three of these were unrelated to the operation or VP shunt. Considering the subjects' mean age of 75 years, vascular events, malignancies, infections, and fractures were not uncommon. Outcomes for those who experienced an SAE were generally unfavorable, as would be expected. In addition, asymptomatic subdural effusion (n = 13) and orthostatic headache (n = 8) were reported as non-serious shunt-related adverse events. These were controlled well by adjustment of valve pressure [24]. Although a considerable number of retrospective case series have been published [26-28], prospective studies using standardized validated outcome measures, which are comparable to the present study, are very scarce. The Dutch NPH study group, in their prospective study, treated 101 patients with iNPH with VP shunt by using fixed pressure valves, and yielded a one-year improvement rate of 59% based on mRS [24]. The group reported quite a high incidence of shunt-related adverse events: transient and persistent subdural effusion in 53% of patients and subdural hematoma requiring surgery in 8%, as well as extraperitoneal displacement, ventricular displacement, disconnection, and infection of the shunt required surgical treatment [25]. Subdural effusion/hematoma was twice as frequent with low-pressure (3-5 cmH_2_O) valves than with medium-pressure (9-11 cmH_2_O) valves [25]. Compared with the Dutch study, the use of the programmable valve and the relatively high average initial valve pressure of 15.5 cmH_2_O based on the pressure-setting scheme in our trial favoured a low incidence of serious CSF over-drainage without affecting shunt effectiveness. Although our results suggest the advantage of CHPV in comparison with fixed-valve shunt systems, head-to-head comparison is needed in randomized controlled trials to draw a conclusion.

Kitagaki *et al*., based on their case-control study involving volumetry of CSF spaces, pointed out that tight high-convexity and subarachnoid spaces support the diagnosis of iNPH [10]. The present study confirmed the diagnostic value of these MRI findings. The Dutch iNPH study group used the sum of the four largest convexity sulci being less than 25 mm on CT as a criterion of tight convexity subarachnoid spaces [24], which is partially compatible with our study. However, axial CT slices are inferior to coronal MRI sections in viewing the high-convexity region. Axial and coronal MRIs accurately detect narrow sulci at the high convexity and midline, which accurately predicts iNPH [29]. Kitagaki *et al*. also reported that distended basal cisterns and Sylvian fissures and focally dilated sulci are features of iNPH [10]. Recently, Ishii et al., in a controlled study using a voxel-based morphometry technique, demonstrated densely compacted parietal gyri in patients with iNPH, as well as ventricular enlargement and Sylvian fissure dilatation [30]. Whereas, in the present trial, candidates were selected only on the basis of ventriculomegaly and tight high-convexity and medial subarachnoid spaces on coronal MRIs, enlarged Sylvian fissures and focally dilated sulci were found in 96% and 29%, respectively. The present prospective cohort study showed enlargement of the Sylvian fissures in the vast majority of patients with iNPH. Enlargement of the Sylvian fissures by retention of CSF is another feature of iNPH. Thus, CSF distributes disproportionately between the superior and inferior subarachnoid spaces. It is morphologically distinguishable from communicating hydrocephalus secondary to subarachnoid hemorrhage or meningitis (secondary NPH) and non-communicating hydrocephalus. We propose the generic term disproportionately enlarged subarachnoid-space hydrocephalus (DESH) for this type of hydrocephalus. The clinical significance of DESH in communicating hydrocephalus is the presence of enlargement subarachnoid spaces specifically in their inferior parts. Enlarged ventricles and large Sylvian fissures in combination with tight high-convexity subarachnoid space should not be misinterpreted as cerebral atrophy.

The present study had several notable limitations that require discussion. The most critical issue is that it lacked a control group. However, as iNPH is a progressive disease, management without surgery would not be allowed for ethical reasons. Another crucial issue is related to the unblinded design of the study: the neurosurgeons and neurologists in charge of the patients' care and evaluation were aware of the treatment, which represents a possible source of performance and detection bias. Thirdly, the central MRI review committee excluded patients who did not fulfil the MRI criteria, which is a source of selection bias. Further, any patients with the MRI criteria but without the symptomatic triad and any patients with the symptoms but without the MRI criteria were not considered. These issues should be taken into consideration before the findings are generalized. Also, in this study, the roles of several diagnostic measures, including the tap test, CSF measurement, and CT cisternography, were examined, and the data related to the long-term management of the shunt system were collected. Information about the additional measures and procedures will be reported in a later paper.

## Conclusions

Our study demonstrated that the MRI-based scheme supports the diagnosis of iNPH. The MRI features of DESH, i.e., tight high-convexity and medial subarachnoid spaces and enlarged Sylvian fissure associated with ventriculomegaly are useful for the diagnosis of iNPH.

## Competing interests

This investigator-initiated study was supported in part by Johnson & Johnson K.K., Nihon Medi-Physics Co. Ltd, and Daiichi Pharmaceuticals Co. The funding sources for the study had no role in the design and conduct of the study, in the collection, analysis, and interpretation of the data, or in the preparation, review, or, approval of the manuscript. Drs. Hashimoto, Ishikawa, and Mori have received honoraria from companies that manufactured the devices discussed in this article, including Johnson & Johnson K.K. (Japan) and Nihon Medi-Physics Co. Ltd. Dr. Kuwana has received honoraria from Johnson & Johnson K.K. (Japan).

## Authors' contributions

All authors had full access to all of the data in the study and take responsibility for the integrity of the data and the accuracy of the data analysis. MH, MI and EM: drafting of the manuscript. NK: critical revision of the manuscript for important intellectual content. All authors have read and approved the final version of the manuscript.
